# Indiana State Medical Society

**Published:** 1870-07

**Authors:** George Sutton, G. V. Woolen

**Affiliations:** President; Secretary


### INDIANA STATE MEDICAL SOCIETY.

This organization commenced its twentieth annual session in Indianapolis, in the lecture room of the Indiana Medical College, corner of Delaware and Court Streets, at 9 o'clock, May 17, 1870.

Dr. Gho. Sutton, of Aurora, took the Chair, and called the Society to order.

Dr. G. V. Woolen, of Indianapolis, the worthy Secretary for several years past, read the following list of officers, members, and delegates who were present:

OFFICERS.

Geo. Sutton, Aurora, President.

G. W. Woolen, M.D., Indianapolis, Secretary.

W. J. Elstun, M.D., Indianapolis, Assistant-Secretary.

PERMANENT MEMBERS.

Dr. C. E. Wright, J. P. Avery, D. H. Oliver, W. J. Elstun, G. W. Woolen, L. D. Waterman, F. S. Newcomer, J. K. Bigelow, W. B. Fletcher, G. W. Mears, J. M. Kitchen, J. H. Woodburn, T. B. Harvey, O. Everts, J. A. Comingor, R. N. Todd, Theo. Parvin, Indianapolis; John Arnold, W. W. Arnold, M. Sexton, John Moffet, Rushville; N. P. Howard, Greenfield; J. W. Powell, Rockfield; Benj. Newland, Bedford; V. Kersey, D. Clarke, J. F. Hibberd, J. R. Weist, R. E. Houghton, Richmond; F. W. Beard, Harrodsburg; F. J. C. Rawlins, A. C. Dillon, N. H. Canady, Knightstown; W. Hobbs, Carthage; F. J. Van Vorhis, Stockwell; J. I. Rooker, Castleton; H. D. Wood, Angola; H. V. Passage, J. H. Helm, Peru; R. G. Graydon, Southport; A. J. McLeod, Burnsville; W. Scott, W. K. Mavity, J. M. Darnell, Kokomo; J. A. Nesbit, Allisonville; W. G. McFadden, London; L. Kern, L. C. Miller, Alto; M. H. Bounel, Lebanon; N. D. Gaddy, Vernon; T. H. Lane, Northfield; J. B. Cox, Frankfort; J. H. Stuart, Spiceland; S. C. Thomas, Milroy; M. H. Harding, Lawrenceburg; Wm. Lomax, J. R. Shively, Marion; J. Pennington, J. E. Swiney, Milton; Wm. Davidson, Madison; J. M. Gray, Noblesville;

James Thompson, Harrison, Ohio; I. N. Parr, Zionville; Irving Sunman, Versailles; I. M. Rosenthal, Fort Wayne; E. H. Crippen, Moscow; J. V. Hoss, Tetersburg.

DELEGATES.

Dr. S. M. Martin, Greenfield; E. N. Tull, Ogden; Edwin Cain, Greensboro; S. A. Troy, Eden; A. B. Casterline, Beechgrove; G. W. H. Kemper, W. S. Andrew, Muncie; I. C. Walker, W. P. Warring, Richmond; H. G. Todd, Danville; W. F. Harvey, Plainfield; C. Robins, Brooklyn; Samuel Schofield, Hamilton; A. P. Mitten, Columbia City; A. W. Davis, Indianapolis; B. S. Woodworth, Fort Wayne.

Permanent members reported:

S. E. Munford, Princeton; E. Mendenhall, Zionsville; W. G. Moore, Indianapolis; D. H. Harding, Batesville.

EVENING SESSION.

Dr. L. D. Waterman, of Indianapolis, moved that the annual address of the President be received this evening at 8 o'clock, and be made the special order for that hour. The motion was agreed to.

CREDENTIALS.—Drs. V. Kersey, of Richmond, W. H. Arnold, of Rushville, and L. D. Waterman, of Indianapolis, were appointed a standing committee of inquiry as to the qualifications of applicants for membership, and upon their favorable report to the Society, a vote of two-thirds of the members present shall be necessary for the election of each one thus recommended.

SECRETARY'S REPORT.—Dr. Woolen, the Secretary, laid before the Society, his annual report in writing. On motion, Drs. Waterman, Johnson, and A. B. Casterline, were appointed a committee to consider and report on the suggestions contained in it.

FIVE MINUTE RULE.—Dr. W. Hobbs, of Carthage, submitted a resolution limiting remarks on any one subject to five minutes, and debarring any member from speaking more than once on the same subject till other members had been heard, unless by unanimous consent.

Dr. J. F. Hibberd, of Richmond, moved to lay it on the table. Agreed to.

Essays.—The President announced the order for reports from committees appointed last year to prepare papers on special subjects.

Dr. J. H. Woodburn, of Indianapolis, in reply to a call for his report, stated that as the Committee on the Presentation of the Symptoms and Treatment of Incipient Insanity, he had endeavored, but in vain, to condense the subject in one paper, consequently he asked to be excused.

Dr. G. W. Mears, of Indianapolis, reported that the committee on the Most Effective Remedies for Arresting Uterine Hemorrhage, will submit a paper at the proper time.

Dr. M. Sexton, of Rushville, announced no report on Tetanus.

Dr. G. V. Woolen, that he would submit a report on Syphilis.

Dr. F. J. Van Vorhis, Stockwell, an essay on the Psychical Influence upon the Organization of Structures, which he proposed to substitute for the subject assigned to him last year, namely: Mental Influences in Disease.

Voluntary Essays.—The President then called for voluntary papers, and the following were reported ready for presentation when called for:

By Dr. W. Hobbs, Carthage, on Surgical Operations in the Removal of Bones of the Skull.

By Dr. C. E. Wright, Indianapolis, on Diseases of the Ear.

By Dr. L. D. Waterman, Indianapolis, on Relapsing Fever.

By R. E. Houghton, Richmond, on Dislocations of the Hip.

By Dr. H. V. Lassage, Peru, on Reduction of Dislocations of the Hip-joint. Also,

By Dr. V. Kersey, Richmond, (in pursuance of a direction by a vote of the Wayne County Medical Society) on the condition of the Medical Staff in the United States Navy.

Public Charities.—Dr. J. R. Weist, Richmond, chairman of the committee appointed last year to consider the propriety of petitioning the State Legislature to constitute and organize a Board of Public Charities, announced that he had a report.

Credentials.—The committee on qualifications of members were duly authorized and empowered to act as a committee on credentials.

Society Seal.—On motion, by Dr. J. I. Rooker, Castleton, a resolution was adopted authorizing the publishing committee and the Secretary, to purchase an appropriate seal for the use of this Society.

Delinquent Members.—Dr. W. B. Fletcher, Indianapolis, submitted a resolution declaring that the names of all members of this Society, who shall fail to pay the annual assessment for three years in succession, after due notification by the Secretary, shall be dropped from the roll.

Dr. Woodburn moved to amend, by making the resolution extend three years beyond the present year. Rejected.

The resolution was then adopted by a rising vote.

In Memory of Dr. J. S. Bobbs.—The Jay County Medical Society transmitted, through Secretary Woolen, resolutions of respect to the memory of Dr. J. S. Bobbs, Indianapolis.

On motion, they were laid on the table for the present.

Dr. Hobbs said: Mr. President—Dr. Bobbs for years was a member of this Society; was once in its chair as our President, and he occupied a prominent position as a medical teacher in this State. I, therefore, think it proper for this Society to do more then pass formal resolutions on this occasion. It occurs to me as proper.

I have had this on my mind without the knowledge that these resolutions would be presented. If in order, I offer the following:—

*Resolved*, That a committee of five be appointed to prepare a paper giving a short biographical sketch of the late Dr. J. S. Bobbs, embracing, also, an account of his professional character and labors, and resolutions of our respect and esteem for him, and present the same to the Secretary of this body, to be embodied in the published transactions.

The resolution was adopted.

Public Charities.—On motion, Dr. Weist read his written report referred to and described above. It embodies a resolution that a committee be appointed by this Society, to petition the State Legislature to enact a law providing for a State Board of Public Charities, similar to those now existing in Massachusetts, Ohio, Illinois, and other States, and to take such other steps as they may deem necessary to secure such legislation.

Dr. Woodburn moved that the report be referred to the Committee on Publication, with a view to its incorporation in the published proceedings of this meeting.

The motion was agreed to.

On motion by Dr. Waterman, the President was authorized to appoint five members who shall constitute the committee contemplated in the resolves embodied in the report of the Committee on Public Charities.

NAVY SURGEONS.—Dr. Kersey, presented the paper on the condition of the Medical Staff in the United States Navy. It refers to the case of Dr. Greene, a Passed-Assistant Surgeon in the U.S. Navy, who was ordered by the Captain to strike from the sick list the name of a seaman, and because he refused, he was court-martialed and reprimanded. He recommended the adoption of a resolution that medical men will keep out of the naval service until guaranteed better treatment.

The resolution embraced in the paper was adopted, and the paper as a whole was referred to the committee for publication in the Society's transactions.

On motion of Dr. R. N. Todd, Indianapolis, a copy was ordered to be transmitted to the American Medical Society, and to the Surgeon-General.

The Society then took a recess for dinner.

AFTERNOON SESSION.

The President resumed the chair at 2 o'clock P.M.

THANKS.—A vote of thanks was passed to the Faculty of the Indianapolis Medical College, for the use of the Principal Lecture Room, in which the sessions of this Society are being held.

NEPHRITIS.—A note, as a report, on the Pathognomic Signs of Nephritis, was read by Dr. J. H. Comingor, of Indianapolis, stating he knew none but what were in the text-books.

Dr. G. W. Mears, of the same city, submitted a well prepared essay on Puerperal Hemorrhage, which was discussed by Drs. Hibberd, Woolen, Lomax, Todd, Wishard. Passage, Harvey, Rooker, Clark, Pennington, Waterman, and Mears.

Professional Advertising.—Dr. J. Thompson, of Harrison, Ohio, presented an argument against the action of the American Medical Association, on the subject of "specialists'" advertising.

A motion being made to refer this paper, Dr. Woodburn would not sit still and see the code of ethics stabbed in this way, without raising his voice in the negative.

Thereupon the motion was so changed as to make this paper the special order for to-morrow afternoon, at two o'clock.

The motion, as modified, was agreed to.

Dr. F. J. Van Vorhis, then read a paper on the Psychical Influence upon the Organization of Structures.

On motion by Dr. Hibberd, it was laid on the table till after the President's address shall be disposed of this evening.

And then came recess till eight o'clock P.M.

Night Session.

Dr. J. F. Hibberd was called to the Chair, and—

The President, Dr. George Sutton, read his address on "Man's Power over Nature; the Science of Medicine as a Means by which He Aids and Controls the Laws of Life."

It was referred to the Committee on Publication, and a vote of thanks was tendered the President for his able and philosophical address.

Dr. Van Vorhis' Paper.—Dr. Van Vorhis' paper, read this afternoon, titled "The Psychical Influence upon the Organization of Structures," having been made the special order for this time in the session, it was taken up and discussed Drs. Kersey, Lomax, Stuart, Rosenthal, Elstun, and Van Vorhis.

The paper was then referred to the Publishing Committee.

Committee on Nominations.—On motion, the President appointed a Committee of five, to nominate officers for the ensuing year, *viz.:* Drs. Clark, Waterman, Moffet, Wishard, and Passage. And then the Society adjourned till to-morrow morning, at eight o'clock.

Second Day.

The President, Dr. Sutton, took the Chair, at 9 o'clock A.M.

The Committee on Credentials presented favorable reports, which were concurred in.

Publication of the Constitution.—Dr. Hibberd offered the following:

*Resolved*, That the Secretaries of this Society be requested to incorporate into the Constitution and By-Laws all the amendments and alterations that have been made since their last publication, including those of this year, and publish the same in the volume of transactions for the current year.

The resolution was adopted.

Amendment to the By-laws.—On motion by Dr. Hibberd, the By-Laws were amended by adding a new section as follows;

Section 6. Every person claiming to be a member of this Society shall, before he be allowed to take part in the proceedings, give his name to the Secretary and pay two dollars.

Practitioner's License.—Secretary Woolen laid before the Society a preamble and series of resolutions adopted by the American Medical Association, at its session in New Orleans, in May, 1869. One of the resolutions requests each State Medical Society to appoint annually one or more Boards of Examiners, whose duty it shall be to meet at suitable times and places, for the examination of all persons, whether graduates of college or not, who propose to enter upon the practice of medicine in their respective States, except such as have been previously examined and licensed by a similar Board, in some other State.

On motion, the President was authorized to appoint a select Committee of three, whose duty it shall be to consider these resolutions and report thereon, if deemed advisable, this afternoon.

The President, thereupon, made Drs. J. F. Hibberd, R. N. Todd, and T. Parvin said Committee.

The President's Address.—On motion, Drs. Hobbs, Harvey, and Passage were named as a Select Committee, to which was referred the President's Annual Address, with instructions to report thereon this afternoon.

Essay on Syphilis.—Dr. Woolen, being called upon by the President, proceeded to read his paper on Syphilis.

On motion by Dr. Clark, it was referred to the Committee on Publication.

Delegate from Ohio.—Dr. Passage introduced Dr. S. S. Gray, a delegate from the Ohio State Medical Society, whose presence was acknowledged by applause.

Caries of the Skull.—Dr. Hobbs' report of an operation for syphilitic caries of the skull being called for, was now read. It gave rise to some little running discussion.

On motion by Dr. Waterman, this paper was laid on the table, and, together with Dr. Woolen's essay on Syphilis, were made the special order for 5 o'clock this afternoon.

Election of Officers.—Dr. Clark, from the Committee on Nomination of Officers for the ensuing year, made the following report:

President—Dr. R. N. Todd, of Indianapolis.

Vice-President—Dr. I. N. Rosenthal, of Fort Wayne.

Secretary—Dr. G. V. Woolen, of Indianapolis.

Assistant-Secretary—Dr. W. J. Elstun, of Indianapolis.

Treasurer—Dr. James H. Woodburn, of Indianapolis.

Librarian—Dr. A. W. Davis, of Indianapolis.

The report was concurred in.

Dislocation of the Hip-Joint.—Dr. Passage being called upon, then read his paper on "Reduction of Dislocation of the Hip-Joint, of eleven days' standing."

Dr. R. E. Haughton, of Richmond, presented a report upon the dislocation of the hip; some of the causes which interfere with reduction; principles of the Flexion method, etc., illustrated by specimens, and drawings on the blackboard.

When he had concluded—

On motion of Dr. Waterman, the Society took a recess till 2 o'clock P.M.

Afternoon Session.

President Sutton, being engaged upon the appointment of Committees, at 2 oclock, on motion, Dr. Hibberd took the chair, and called the Society to order.

Professional Advertising.—On motion of Dr. Woodburn, the special order for this hour, Dr. Jas. Thompson's paper, concerning the publication of cards, circulars, or advertisements by specialists, was referred to the Committee on Ethics.

Dislocation of Hip-Joints.—The papers pending at the hour of recess for dinner were referred to the Committee on Publication, on motion of Dr. Harvey.

Diseases of the Ear.—Dr. C. E. Wright, of Indianapolis, being called upon for his paper on Purulent Aural Catarrh, came forward and read it. Referred to the Committee on Publication.

Dr. Rooker offered the following resolution, which was adopted:

*Resolved*, That this Society recommend the profession of the State to subscribe for, and contribute papers to, the *Indiana Medical Journal.*

The President *pro tem.*, in behalf of President Sutton, announced the Committee to prepare and submit to the State Legislature a petition for the enactment of a law providing for a State Board of Public Charities, namely: Drs. Ryland T. Brown, Patrick H. Jameson, James H. Woodburn, Orpheus Evarts, and James F. Hibberd.

Dr. Lomax, in behalf of Treasurer Lyons, unavoidably absent, submitted the Treasurer's Annual Report, which was referred to the Committee on Finance.

Dr. E. Mendenhall, of Zionville, then read his paper, titled "The Utility of Ergot in Facilitating Labor."

On motion, this paper was referred to the Committee on Publication.

Financial.—Dr. Harding, from the Committee on Finance, reported the accounts of the Secretary and Treasurer correct, and recommended an assessment of $2 per member, to meet current expenses.

The report was concurred in.

Thanks to the Daily Papers.—Dr. Thad. M. Stevens offered the following:

*Resolved*, That the thanks of this Society are due, and are hereby tendered to, the daily press of this city, and especially to the *Journal* and *Sentinel*, for their more than ordinary accurate and full reports of our proceedings.

The resolution was adopted *nem. con.*

Practitioner's License.—Dr. Hibberd, from the Special Committee thereon, returned the preamble and resolutions of the American Medical Association, touching the admission of new men into the ranks of practicing physicians, reported it clearly impossible to find time for the deliberate consideration of the subject, in the few remaining hours of this meeting, and therefore the Committee recommend the reference of the resolutions to a Special Committee, for digestion, with instructions to report next year.

On motion, the report was referred back to the same Committee, with instructions to consider the subject and report at the next annual meeting.

The President's Annual Address.—Dr. Hobbs, from the Special Committee on the President's Address, submitted a report, recommending that Dr. George Sutton himself be requested to report a paper to the next year's meeting on "The Nature, Pathology, and Treatment of Milk Sickness, and its relation to what is called 'Trembles,' in Inferior Animals."

Also, that some one be appointed to report at the same time Biographical Sketches of Physicians who have died in the State. Dr. J. H. Stuart, Spiceland, was appointed.

Also, some one to prepare a report on the changes, if any, in the types, which the diseases of the State are undergoing.

The Chair appointed Dr. G. W. Mears, Indianapolis.

Also, that a Committee of one from each Congressional District be appointed to collect and collate facts relating to the health of their different localities, medical statistics, etc.

Subsequently the Society selected the said Committee, *viz.:*

1st District—Dr. Daniel Morgan, Evansville.

2d District—Dr. Wm. Clapp, New-Albany.

3d District—Dr. A. G. Boynton, Elizabethtown.

4th District—Dr. S. M. Martin, Greenfield.

5th District—Dr. Henry G. Todd, Danville.

6th District—Dr. T. H. Rice.
7th District—Dr. J. H. Adams.
8th District—Dr. W. K. Mavity, Kokomo.
9th District—Dr. N. H. Kennedy, Knightstown.
10th District—Dr. A. D. Wood.
11th District—Dr. Lewis Humphreys, South Bend.

CERTIFICATES OF MEMBERSHIP.—Dr. Kersey, from the Special Committee on the Secretary's report, recommended the granting of certificates, signed by the President and Secretary, and sealed with the seal of the Society, to any member in good standing about to remove from the State.

The report was concurred in.

PERMANENT MEMBERS.—The following additional names were recorded. R. C. Moore, Belleville; C. N. Blount, Tipton; John Rea, Newcastle; George Sutton, Aurora; I. J. Adams, North Salem; Wood Cook, Pendleton; J. M. Wishard, T. B. Noble, Greenwood; B. Ward, P. H. Jameson, Indianapolis; W. Lockhart, W. J. Hoadley, Danville; M. V. B. Newcomer, Tipton.

DELEGATES.—H. M. Minesinger, Sulphur Springs; J. W. Badger, Fremont; T. C. Rich, Indianapolis; J. J. Saville, Kokomo; G. M. Adams. Frankfort; G. W. Leutz, Lawrenceburg.

THANKS TO DR. KITCHEN.—Dr. Harvey offered the following resolution:

*Resolved*, That this Society accept the offer of Dr. J. M. Kitchen to furnish the complete files of the transactions; and we hereby tender our thanks to him for the same, and instruct the Librarian to have the same preserved in the Library.

The resolution was adopted.

LIST OF DELEGATES.—Dr. Clark, from the Committee on Nominations, recommended as delegates to the American Medical Association, Drs. V. Kersey, Thad. M. Stevens, G. Sutton, M. H. Harding, W. Scott, W. K. Mavity, J. P. Avery, J. F. Hibberd, J. R. Weist, A. P. Mitten, H. V. Passage, B. Newland, J. I. Rooker, W. Lockhart, J. C. Walker, S. C. Tomlinson, N. D. Gaddy.

The report was concurred in.

Subsequently, on motion of Dr. Clark, it was

*Ordered,* That any gentleman may become a delegate by reporting his name to the Secretary, previous to the publication of the transactions of this meeting in book form.

On motion by Dr. Elstun, it was

*Resolved,* That any member of this Society who desires to attend the Ohio State Medical Society be furnished a certificate as delegate.

The Next Annual Meeting.—The President *pro tem.* suggested that as the American Medical Association meets next year in San Francisco, California, on the 2d Tuesday in May, Delegates from this Society could not return to our meeting unless the time be changed.

Thereupon, Dr. Passage moved that the next meeting of this Society be held on the 2d Tuesday in April next.

Dr. Weist moved to amend by fixing the time for the 2d Tuesday in June, 1871, at 9 o'clock A. M.

The amendment was agreed to.

The motion as amended was then adopted.

Relapsing Fever.—Upon a call from the President *pro tempore,*

Dr. Waterman read his voluntary paper on Relapsing Fever, and requested that it should not be referred for publication, as it was prepared in great haste, because he expected a dearth of reports this year.

It gave rise to a brief discussion, in which Drs. Clark, Van Vorhis, Rooker, and others took part, in which regrets were expressed that Dr. Waterman had not put his report in such shape as that he would consent to its publication in the transactions of the Society.

On motion by Dr. Kersey, the thanks of the Society were tendered Dr. Waterman for his paper.

Standing Committees.—The President announced the following standing committees for the ensuing year:

On Prize Essays—Drs. T. Parvin, G. W. Mears, and D. Clark.

On Ethics—Drs. T. B. Harvey, F. H. Woodburn, James Thompson, J. M. Kitchen, and C. N. Blount.

On Arrangements—Drs. L. D. Waterman, A. W. Davis, W. W. Foley, J. A. Comingor and W. G. Moore.

On Finance—Drs. W. Lockhart, F. J. Van Vorhis, H. V. Passage, W. K. Mavity, and J. Moffet.

On Publication—Drs. G. V. Woolen, W. J. Elstun, L. D. Waterman, C. E. Wright, and W. B. Fletcher.

Special Essayists.—Were also announced, to wit:

Dr. M. H. Harding, Lawrenceburg, on Hydrate of Chloral.

Dr. J. F. Hibbard, Richmond, on the Progress of Medicine.

Dr. W. B. Fletcher, Indianapolis, on the formation of the Placenta.

Dr. John Moffet, Rushville, on such subject as he may choose.

Dr. Dugan Clark, Richmond, on Chloroform in Obstretrics.

Dr. W. Hobbs, Carthage, on Alcoholic Stimulants in the Treatment of Disease.

Dr. C. E. Wright, Indianapolis, on Diseases of the Eye.

Dr. J. R. Weist, Richmond, on a subject to be selected.

Dr. W. Lomax, Marion, to select his own subject.

Dr. S. C. Thomas, Milroy, on Hemorrhage in Typhoid Fever.

Dr. W. J. Elstun, on Therapeutics of Bromide of Potassium.

Dr. B. S. Woodworth, Fort Wayne, to select his own subject.

Dr. Theo. Parvin, Indianapolis, on Fibrous Tumors of the Uterus.

Dr. T. B. Harvey, Indianapolis, on Laceration of the Perineum.

Dr. C. N. Blount, Tipton, subject to be selected.

Dr. V. Kersey, Richmond, on Inflammation.

Syphilis.—The President *pro tem.* announced the subject matter (the two papers on Syphilis) made the special order for discussion at five o'clock.

Drs. Woolen, Harding, Seville, Hobbs, Waterman, and Haughton made remarks on the subject.

The paper of Dr. Hobbs was referred to the Committee on Publishing. Dr. Woolen's already having been referred.

Thanks to the President.—Dr. Waterman moved a vote of thanks to our worthy President for discharging his duties so acceptably during the present session.

The motion was agreed to.

President Sutton acknowledged the compliment in a few pertinent remarks.

The Society adjourned till the second Tuesday of June, 1871.

GEORGE SUTTON, *President.*

G. V. WOOLEN, *Secretary.*

